# Incidence, clinical profile, and risk factors for serious bacterial infections in children hospitalized with fever in Ujjain, India

**DOI:** 10.1186/s12879-020-4890-6

**Published:** 2020-02-21

**Authors:** Ashish Pathak, Radika Upadhayay, Aditya Mathur, Sunil Rathi, Cecilia Stålsby Lundborg

**Affiliations:** 1 Department of Paediatrics, Ruxmaniben Deepchand Gardi Medical College, Ujjain, Madhya Pradesh 456010 India; 2 Department of Women and Children’s Health, International Maternal and Child Health Unit, Uppsala University, Uppsala, Sweden; 3 Department of Global Public Health, Health Systems and Policy: Medicines Focusing Antibiotics, Karolinska Institutet, Stockholm, Sweden

**Keywords:** Fever, Serious bacterial infection, Children, Risk factors

## Abstract

**Background:**

Fever is a cause for concern for both parents and the treating pediatrician and a common reason for antibiotic overuse. However, the proportion of children hospitalized for fever with serious bacterial infection (SBI) is uncertain. We aimed to evaluate the epidemiological, clinical, hematological, and biochemical risks for SBI among the children admitted with fever.

**Method:**

This prospective study was conducted in a rural teaching hospital in India on consecutive children, aged 3 months–12 years, presenting with fever 100 °F (37.7 °C) or higher. The presence of SBI was confirmed with one of the following criteria: (a) a positive blood culture; (b) roentgenographically confirmed pneumonia with high titres of C-reactive protein; (c) a culture-confirmed urinary tract infection; (d) enteric fever diagnosed clinically in addition to either a positive blood culture or high Widal titers; and (e) meningitis diagnosed clinically in addition to either a positive blood culture or cerebrospinal fluid culture. A predefined questionnaire was filled.

**Results:**

A total of 302 children were included in the study, out of which 47% (95% CI 41.4–52.7%) presented with SBI. The factors associated with confirmed SBI in bivariate analysis were history of previous hospitalization, history of chronic illness, history of medication in the previous 1 week, a partially immunized child, history of common cold, moderate-grade fever, toxic look, significant lymphadenopathy, absence of BCG scar, delayed development, irritability, breathlessness, respiratory distress, poor feeding, significant weight loss, suspected urinary tract infection, hyponatremia, hypokalemia, and abnormal leucocyte count. The final generalized logistic regression model revealed partially immunized child (RR 4.26), breathlessness (RR 1.80), weight loss (RR 2.28), and suspected urinary tract infection (RR 1.95) as risk factors for the increased risk of SBI.

**Conclusion:**

The study identified multiple risk factors for SBI. Pediatricians can be made aware of these risk factors. Further studies are warranted to identify age-specific risk factors for SBI because most clinicians depend on clinical signs and symptoms to identify SBI.

## Background

Fever is a common clinical symptom in children and is one of the leading causes for medical consultation and hospital admissions [1]. Globally, 5.4 million children die before the age of 5 years and approximately 50% of the mortality is caused by infectious diseases, many of which present with fever [2].

The underlying infectious disorder causing fever in children can range from mild and self-limiting illness such as upper respiratory tract infection to more serious viral and bacterial illnesses [3]. Distinguishing between the benign self-limiting illness that is manageable at home and those that require hospitalization is the primary challenge for pediatricians and primary care physicians. The diagnosis remains challenging because a wide range of infections can cause fever in a child. The diagnostic challenge is exacerbated in resource-poor settings in terms of laboratory infrastructure and human capacity [4]. The overlapping manifestations of serious bacterial infections (SBIs) with other viral, fungal, parasitic and systemic inflammatory conditions and neoplasms further complicates diagnosis. The overreliance on clinical diagnosis is one of the reasons for irrational use of antibiotics [5].

In the state of Madhya Pradesh in India, where the present study was conducted, National Family Health Survey-4, undertaken in the year 2015–2016, reported that approximately 71% of the children less than 5 years of age were taken to a health facility for complaints of fever in 2 weeks preceding the survey [6]. Studies from India have reported febrile illness due to SBIs, such as pneumonia [7], typhoid fever [8], urinary tract infections (UTIs) [9], blood-stream infections [10], and meningitis [11]. However, as per our knowledge, there is lack of published studies from India on epidemiological or clinical predictors of SBIs among children hospitalized with fever. Clinical prediction rules complement practice guidelines and help physicians improve clinical decision-making [12, 13]. This study aimed to determine the incidence of SBIs in febrile children admitted in a rural hospital in India and to determine the clinical profile and risk factors associated with SBIs.

## Methods

This prospective cohort study was done during the 12 months period from July 2015 to June 2016.

### Study setting

This study was done in the Pediatrics ward of C.R. Gardi Hospital (CRGH), which is a teaching hospital attached to R.D. Gardi Hospital (RDGMC), Ujjain, in the state of Madhya Pradesh, in central India. Department of Pediatrics, RDGMC has 90 beds, distributed in two wards, out of a total of 630 beds in CRGH. A six-bedded pediatric intensive care unit is also attached to the department.

### Provider characteristics

During the study period, the Department of Pediatrics had three units, each having 30 beds, distributed in two wards. Each unit had at least three residents and teaching faculty consisting of a professor, an associate professor, and an assistant professor. The experience of each professor was a minimum of 8 years and that of associate professor and assistant professor at least 5 and 3 years respectively.

### Selection of the participants

Consecutive patients aged between 3 months to 5 years, admitted for fever and having an axillary temperature of 100 °F (37.7 °C) or more taken with mercury thermometer at the time of admission were included in the study. Children admitted with fever but having chronic co-morbidities: malignancy, renal failure, hepatic failure, congestive cardiac failure, and bone marrow aplasia, children on immunosuppressive drugs such as steroids, and HIV positive children were not included in the study. Children transferred-in with fever from other hospitals with a diagnosed bacterial infection or a laboratory result suggestive of bacterial infection were not included, as it would not have allowed an independent clinical assessment of cases. Children with osteomyelitis, cellulitis and patients with surgery and trauma were not included as these children are routinely admitted in wards other than pediatrics.

### Data collection

All the admitted patients were screened for eligibility by the pediatric resident on-call as soon as possible after admission. The details of the study were discussed with the mother or caregiver accompanying the child fulfilling the inclusion criteria and a written consent obtained. After obtaining consent, a standardized pre-defined questionnaire containing clinical history and physical examination was filled in. The questionnaire also contained epidemiological, clinical, hematological and biochemical parameters of the cases. Patients were provided immediate routine or intensive care as per the department protocol. A senior consultant examined all patients within 24 h of admission. Investigations were done at the discretion of the senior consultant.

### Definitions

The definitions used in the study are provided in supplementary Table 1.

### Outcome measure

The unit of analysis was the child and not the number of febrile episodes. The primary outcome was proportion of children having SBI among the children presenting with fever. The presence of SBI was confirmed by presence of at least one of the following criteria: 1) blood culture positive; 2) a child was considered to have bacterial pneumonia: if the child presented with breathlessness and had blood culture positive or if along with breathlessness, chest X-ray showed consolidation and C-reactive protein (CRP) value was more than 1000 μg/dl [14]. X-ray chest were reported by a radiologist not part of the study; 3) a child was considered to have UTI if in a toilet trained child with suspected UTI midstream clean catch urine sample was culture positive and in non-toilet trained children with suspected UTI urine obtained by transurethral bladder catheterization was culture positive; all children were screened for UTI using urine microscopy done on a spun urine sample within 2 h of collection, with 5 or more white blood cells per high power field. 4) a child was diagnosed to have enteric fever when presentation was fever with malaise, headache, abdominal discomfort, coated tongue and Widal test positive with somatic antigen (O) and flagellar antigen (H) titers greater than 320 or blood culture was positive for *S. typhi;* 5) a child was considered to have bacterial meningitis in presence of clinical features suggestive of meningitis, with either blood culture and/or cerebrospinal fluid culture was positive. A fever was labeled as “no confirmed bacterial infection” when the above diagnosis was ruled out, thus viral fever was a diagnosis of exclusion. The secondary outcome was to determine the epidemiological, clinical, hematological, and biochemical risk factors associated with SBI.

### Laboratory methods

Two milliliter (ml) of blood was obtained for hematological and biochemical investigations and 3–4 ml to 10 ml for blood culture according to child’s age. The following investigation were done for all children included in the study: a) complete blood count done using five-part automated coulter counter using fluorescence flow cytometry (XS-800i, Sysmex India Pvt. Ltd., India). Peripheral smear examination was done for leucocyte morphology, immature to mature cell ratio and toxic granules, malaria parasite (thick and thin smear) and red cell morphology; b) a quantitative C- reactive protein (CRP) (Vitros CRP slides, Vitros 250 Chemistry Analyzer, Ortho Clinical Diagnostics, Johnson & Johnson, USA); c) serum electrolytes: serum sodium, potassium, and calcium (Vitros 250 Chemistry Analyzer, Ortho Clinical Diagnostics, Johnson & Johnson, USA); d) Widal test was done using slide and tube agglutination test (Febrile Antigen Set, Span Diagnostic Ltd., India); e) blood and cerebrospinal fluid (CSF) culture was collected under sterile conditions in Bactec Peds Plus/F vial® and pathogens were isolated using the automated BacT/ALERT system (bio-Mérieux, Inc., Marcy l’Étoile, France); e) Urine cultures were done for urine samples having with 5 or more white blood cells per high power field on urine microscopy-urine sample was inoculated into the Blood Agar plate and Mac-Conkey agar plate using the semi quantitative method and incubated aerobically at 37 °C for 24 h. A 0.5 McFarland suspension was prepared from pure culture of uro-pathogens in a nutrient broth and inoculated on Muller-Hinton agar. Antimicrobial susceptibility testing for blood and urine cultures was done using the Kirby Bauer disk diffusion method and results interpreted according to 2018 Clinical and Laboratory Standards Institute guidelines [15]. Extended-spectrum β-lactamase (ESBL) production was detected using a double-disc synergy test [16]. Multidrug-resistant isolates were defined as isolates having co-resistance to at least three antibiotic groups [17]. For all children with probable intrathoracic tuberculosis we collected gastric aspirate and induced sputum samples on 2 consecutive days. All samples were subjected to smear examination after Ziehl-Neelsen staining. An aliquot of each sample was tested using Xpert MTB/RIF assay.

### Sample size calculation

The proportion of SBI is known to vary between 24 to 40%, according to geographical area and the age of children [18, 19]. We choose a proportion of 30% from above studies to calculate the sample size. Sample size calculation was done to detect at least 15% difference around proportion of 0.30, with a power of 90 and two-sided alpha of 0.05. The estimated minimum sample size needed was 230 children. Assuming that 20% children will not be able to complete investigations for fever we increased the sample size by a similar proportion to 276 (230 × 0.2).

### Data analysis

Data was entered in EpiData Entry (Version 3.1, Epi Data Software Association, Odense Denmark) and statistical analyses was performed using Stata (Version 13.0 Statacorp. Texas, USA). For continuous variables range, mean and standard deviation (SD) are presented. Categorical independent variables were investigated using Pearson chi-square with the dependent variable being SBI (yes/no). In case of cell count less than five or at least one cell-count equal to zero, the variable was excluded for further analyses. Pearson Chi-square test was used to test for each risk factor’s association with SBI.

A generalized linear regression model was used to examine the association of independent risk factors responsible for SBI. The independent risk factors were compared between neonates with and without SBI (binary outcome variable). The adjusted relative risk (RR) of SBI was calculated using multivariate predicted marginal proportions for logistic regression models and included the following independent variables as covariates: sex—male versus female; age—in months as continuous; partially immunized child—yes versus no; breathlessness—yes versus no; weight loss—yes versus no; and suspected UTI—yes versus no. The means along with the associated 95% confidence intervals (CI) and *P* values were reported from GLMs. A *P* value of < 0.05 was considered significant in the final model. For the final model, model discrimination was done using C-statistics.

### Ethical considerations

The Institutional Ethical Committee approved the study (Approval number IEC Ref No 461/2015). The procedures followed were in accordance with the ethical standards set by the institutional ethics committee and with the Declaration of Helsinki.

## Results

**Fig. 1:**
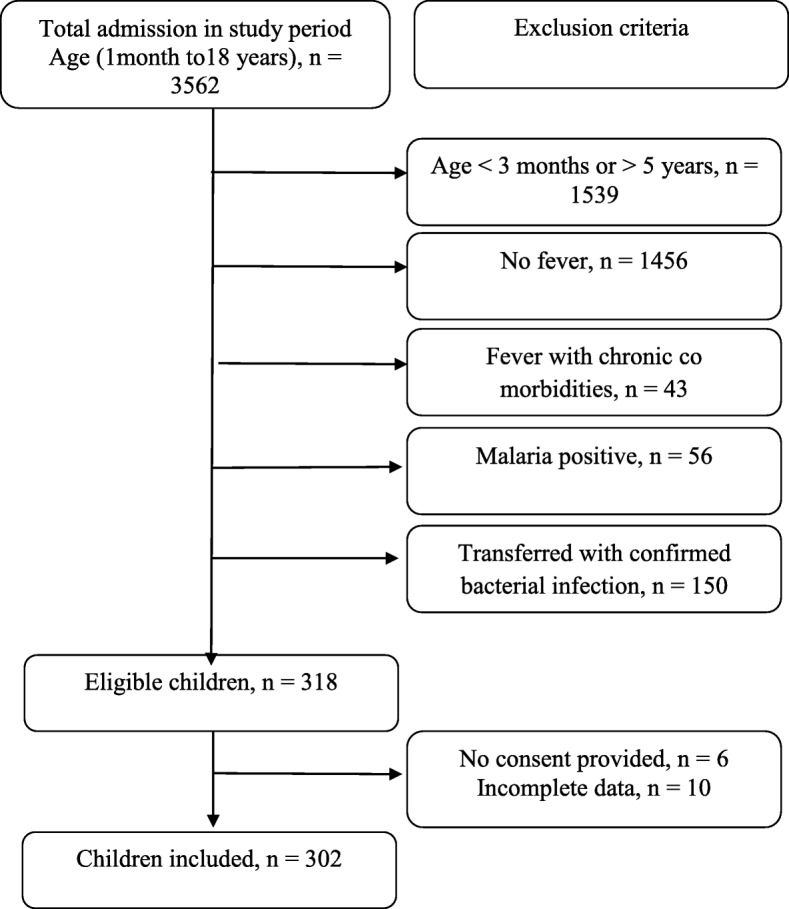
Study flow chart showing recruitment process of patients presenting with fever

The total number of children admitted in the Pediatric ward during the study period, patients presenting with fever, number of patients excluded and reasons of exclusion, are shown in Fig. 1.

### Causes of SBI

The distribution of diagnosis of 302 children presenting with fever according to presence or absence of SBI is shown in Table 1. Out of 302 febrile children, 142 children had SBI. The incidence of SBI was thus, 47% (95% CI 41.4–52.7%). The SBI based on all positive cultures (blood, urine and CSF) was 30% (*n* = 92) and based on only positive blood culture was 26% (*n* = 78); but varied with clinical diagnosis. The most common diagnosis was bronchopneumonia (20%) with 93% SBI rate. Meningitis and enteric fever had high SBI proportion of 58 and 55%, respectively. The SBI in bronchopneumonia was diagnosed in 57 children. The diagnosis was based on positive blood cultures in 33% (*n* = 19/57) and the remaining 67% (*n* = 38/57) were diagnosed as having SBI based on positive chest X-ray and CRP > 1000 μg/dl. Overall X-ray chest showed consolidation in 93% (*n* = 53/57) of cases of bronchopneumonia. The remaining cases were diagnosed based on breathlessness and positive blood culture (Table 1). In enteric fever SBI was diagnosed in 22 children. The diagnosis was based on clinical symptoms and a positive blood culture in 45% (*n* = 10/22) and in the remaining 55% (*n* = 12/22) based on clinical symptoms and positive Widal test (Table 1). Nearly one-fourth of suspected UTI were diagnosed SBI based on urine culture (Table 1). In our study 94% (*n* = 29/31) of the children presenting with severe acute malnutrition (SAM) and fever had confirmed SBI (Table 1). Majority (86%) had evidence of bronchopneumonia, followed by sepsis (7%), UTI (5%) and meningitis (2%). Septicemia was diagnosed in 3 out of 9 cases (33%) of SBI due to UTI, 9 out of 57 cases (16%) of SBI due to bronchopneumonia, 2 out of 29 cases (7%) of SBI due to SAM, and 2 out of 22 cases (4.5%) of SBI due to enteric fever. Two children died (*n* = 2/302, 0.6%) during the study period, both had urosepsis.

**Table 1:** Distribution of diagnosis of 302 children presenting with fever according to presence or absence of serious bacterial infection

Final Diagnosis	Total (n)	Outcome variable Serious bacterial infection		Culture positive	Commonest isolated 2 organism
	302 (%)	No (%)^a^	Yes (%)^a^	(n)	
Bronchopneumonia^b^	61(20)	4(7)	57^b^(93)	19(33)	*P. aeruginosa n* = 4, *K. pneumoniae* *n* = 3,
Viral fever (No confirmed bacterial infection)	52(17)	52 (100)	0	0	–
Urinary tract infection	38(13)	29 (76)	9 (24)	9(100)	*E. coli* n = 7, *P. aeruginosa* n = 2
Severe acute malnutrition	31(10)	2(61)	29(94)	29(100)	*E. coli n* = 13, *S. aureus* n = 9
Febrile convulsion	25(8)	24(96)	1(4)	1(100)	*S. aureus* n = 1
Enteric fever	40(13)	18(45)	22(55)	10(45)	*S.* Typhi n = 10
Bronchiolitis	14(5)	14(100)	0(0)	0	–
Bacterial Meningitis	12(4)	5(42)	7(58)	7(100)	*S. pneumonia* n = 5, *S. aureus* n = 2
Failure to thrive	10(3)	1(10)	9(90)	9(100)	*P. aeruginosa* n = 3, *K. pneumoniae* n = 2
Upper respiratory tract infection	8(2.5)	8(100)	0(0)	0	–
Infective endocarditis in cardiac disease	5(1.5)	0(0)	5(100)	5(100)	*S. pneumonia* n = 3, *S. epidermidis* n = 2,
Miliary tuberculosis	4(1.3)	1(25)	3(75)	3(100)	*P. aeruginosa* n = 3
Dysentery	2(0.7)	2(100)	0(0)	0	–

%-column percentage, %^a^-row percentage, ^b^ 38 cases were diagnosed X-ray chest positive and CRP > 1000 μg/dl. No respiratory samples were cultured

### Results of blood culture, CSF and urine cultures and antibiotic susceptibility testing

The overall culture positivity rate was 30% (92/302), with Gram-negative preponderance (65%). The most common Gram-negative bacteria isolated were *Escherichia coli* (*n* = 25), *Pseudomonas aeruginosa* (*n* = 17), followed by *Salmonella typhi* (*n* = 10) and *Klebsiella pneumoniae* (*n* = 5). The most common Gram-positive bacteria was *Staphylococcus aureus* (n = 17), followed by *Streptococcus pneumoniae* (*n* = 7). The in vitro antibiotic susceptibility pattern of the Gram-negative and Gram-positive pathogens are presented in Supplementary Table 2 and Table 3, respectively. High resistance for beta-lactam antibiotics (range 66–100%), ciprofloxacin (40–100%) and all orally used antibiotics was observed in three common Gram negative bacteria viz. *Escherichia coli*, *Pseudomonas aeruginosa* and *Klebsiella pneumoniae*. The ESBL rates were 75% for *E.coli* and 68% for *Klebsiella*. The MDR rates were 28% for *E.coli* and 33% for *Klebsiella*.

### Factors associated with SBI- bivariate analysis and multivariate logistic regression analysis

The most common age group of children presenting with fever were those between 13 and 36 months (*n* = 123, 41%) with 91(51%) boys and 51(42%) girls. Most (74%) children belonged to lower-middle socio-economic class (Table 2). The duration of hospital-stay of the febrile children having SBI was longer. It was also calculated that with each day increase in stay beyond 7 days, the risk of *p*resence of SBI increased by 1.57 times (95%CI 19–2.07; *P* value< 0.001). The factors on history and physical examination that were significantly associated with SBI on bi-variate analysis are shown in Table 3. The laboratory findings associated with SBI were hyponatremia, hypokalemia and abnormal leucocyte count and are presented in Table 4.

**Table 2:** Association of serious bacterial infection with co-factors related to demographic, socioeconomic factors, and past history in 302 children admitted with fever in Ujjain, India

Independent Variable		Outcome variable Serious bacterial infection				
	Total(%)# *n* = 302	No (%) *n* = 160	Yes (%) *n* = 142	RR	95%CI	*P* value
Sex						
Male	180(60)	89(49)	91(51)	0 .826	0.64–1.06	0.143
Female	122(40)	71(58)	51(42)			
Age in months						
Infant (3mo-12mo)	106(35)	53(50)	53(50)	R	R	–
Toddler (>12mo-36 mo)	123(41)	68(55)	55(45)	0.808	0.48–1.36	0.425
Pre-school (> 36 mo-60 mo)	73(24)	39(53)	34(47)	0.871	0.47–1.58	0.652
Socioeconomic status						
Upper middle	70(23)	37(53)	33(47)	R	R	–
Lower middle	223(74)	119(53)	104(47)	0.97	0.57–1.67	0.941
Lower	9(3)	4(44)	5(56)	1.40	0.34–5.66	0.636
H/o hospitalization (past 1 mo)						
No	267(88)	157(59)	110(41)	2.21	1.86–2.64	< 0.001
Yes	35(12)	3(9)	32(91)			
H/o chronic illness						
No	295(98)	159(54)	136(47)	1.85	1.34–2.57	< 0.001
Yes	7(2)	1(14)	6(86)			
H/o any medication (last one week)						
No	266(88)	155(58)	111(42)	2.06	1.70–2.50	< 0.001
Yes	34(11)	4(12)	30(88)			
Immunization status						
Fully immunized	107(35)	97(91)	10(9)	7.24	3.98–13.17	< 0.001
Partially immunized	195(65)	63(32)	132(68)			
H/o malaise						
No	268(89)	146(54)	122(46)	1.29	0.94–1.76	< 0.001
Yes	34(11)	14(41)	20(59)			
H/o fatigue						
No	255(84)	139(56)	116(45)	1.21	0.90–1.62	0.186
Yes	47(16)	21(45)	26(55)			
H/o lethargy						
No	284(94)	152(54)	132(46)	1.19	0.77–1.84	0.418
Yes	18(6)	8(44)	10(56)			
H/o restlessness						
No	298(99)	158(53)	140(47)	1.06	0.39–2.85	0.902
Yes	4(1)	2(50)	2(50)			
H/o common cold						
No	193(64)	128(66)	65(34)	2.09	1.66–2.64	< 0.001
Yes	109(36)	32(29)	77(71)

**Table 3:** Association of serious bacterial infection with signs and symptoms at presentation of 302 children admitted with fever in 302 children admitted with fever in Ujjain, India

Independent Variable		Outcome variable Serious bacterial infection				
	Total(%)# *n* = 302	No (%) *n* = 160	Yes (%) *n* = 142	RR	95%CI	*P* value
Fever at admission						
Low grade fever (100.1–102.2 °F) (37.8–39 °C)	134(44)	86(64)	48(36)	R	R	–
Moderate fever (102.3–104 °F) (39.1–40 °C)	154(51)	67(44)	87(56)	2.32	1.44–3.74	< 0.001
High grade fever (104.1–106 °F) (40.1–41.1 °C)	14(5)	7(50)	7(50)	1.79	0.593–5.41	0.301
Toxic look						
No	118(39)	79(67)	39(33)	1.69	1.27–2.25	< 0.001
Yes	184(61)	81(44)	103(56)			
Significant lymphadenopathy						
No	245(81)	145(59)	100(41)	1.80	1.45–2.24	< 0.001
Yes	57(19)	15(26)	42(74)			
BCG scar						
Present	249(82)	151(61)	98(39)	2.10	1.73–2.56	< 0.001
Not present	53(18)	9(17)	44(83)			
Generalized swelling						
No	297(98)	159(54)	138(46)	1.72	1.09–2.71	0.019
Yes	5(2)	1(20)	4(80)			
Delayed development						
No	284(94)	158(56)	126(44)	2.00	1.62–2.46	< 0.001
Yes	18(6)	2(11)	16(89)			
Irritability						
No	241(80)	143(60	98(41)	1.77	1.42–2.20	< 0.001
Yes	61(20)	17(28)	44(72)			
Seizures						
No	262(87)	134(51)	128(49)	0.71	0.46–1.11	0.137
Yes	40(13)	26(65)	14(35)			
Breathlessness						
No	214(71)	142(66)	72(34)	2.36	1.90–2.93	< 0.001
Yes	88(29)	18(20)	70(80)			
Respiratory distress						
No	220(73)	140(64)	80(36)	2.07	1.67–2.57	< 0.001
Yes	82(72)	20(24)	62(76)			
Poor feeding						
No	250(83)	147(59)	103(41)	1.82	1.46–2.25	< 0.001
Yes	52(17)	13(25)	39(75)			
Weight loss						
No	263(87)	157(60)	106(40)	2.29	1.92–2.72	< 0.001
Yes	39(13)	3(8)	36(92)			
Vomiting						
No	251(83)	131(52)	120(48)	0.90	0.64–1.26	0.554
Yes	51(17)	29(57)	22(43)			
Gastrointestinal complains						
No	258(85)	132(51)	126(49)	0.74	0.49–1.12	0.159
Yes	44(15)	28(64)	16(36)			
Suspected UTI						
No	173(57)	133(77)	40(23)	3.41	2.56–4.55	< 0.001
Yes	129(43)	27(21)	102(79)			

%#-column percentage, %- row percentage, BCG- Bacillus Calmette–Guérin, UTI-Urinary Tract Infection

**Table 4:** Association of serious bacterial infection with laboratory findings at presentation of 302 children admitted with fever in Ujjain, India

Independent Variable		Outcome variable Serious bacterial infection				
	Total(%)# *n* = 302	No (%) *n* = 160	Yes (%) n = 142	RR	95%CI	*P* value
Hyponatremia						
No	255 (84)	144 (56)	111 (44)	1.51	1.18–1.94	0.001
Yes	147 (16)	16 (34)	31 (66)			
Hypernatremia						
No	185 (61)	96(52)	89 (48)	0.94	0.73–1.20	0.636
Yes	117 (39)	64 (55)	53 (45)			
Hypokalemia						
No	267 (88)	149 (56)	118 (44)	1.55	1.19–2.01	0.001
Yes	35 (12)	11 (31)	24 (69)			
Hyperkalemia						
No	272 (90)	143 (53)	129 (47)	0.91	0.56–1.40	0.679
Yes	30(10)	17 (57)	13 (43)			
Hypocalcemia						
No	284 (94)	154 (54)	130 (46)	1.45	1.02–2.06	0.035
Yes	18 (6)	6 (33)	12 (67)			
Abnormal leucocyte count						
No	22 (7)	18 (82)	4 (18)	2.71	1.10–6.62	0.029
Yes	280 (93)	142 (51)	138 (49)			

%#-column percentage, %- row percentage

The details of the final model are presented in Table 5. The ROC of the final fitted model was 0.964, which reflects excellent model fit. In the final logistic regression model the following variables were found to be statistically significant associated with SBI: unimmunized versus partly immunized status (RR 4.26), breathlessness (RR 1.80), presence of weight loss (RR 2.28), and suspected UTI (RR 1.95).

**Table 5:** Multivariate logistic regression model for serious bacterial infection for 302 children admitted with fever in Ujjain, India

Independent Variable	Outcome variable Serious bacterial infection		
	adjusted RR	95%CI	*P* value
Sex	0.87	0.62–1.24	0.464
Age in month (continuous)	1.00	0.99–1.01	0.262
Partly immunized child	4.26	2.17–8.37	< 0.001
Breathlessness	1.80	1.25–2.61	0.001
Weight loss	2.28	1.13–3.40	< 0.001
Suspected UTI	1.95	1.32–2.88	0.001

## Discussion

This is the first study from central Indian province of Madhya Pradesh that reports the SBI rates among children. The study addresses a significant concern that general *practitioner* and pediatricians face while treating children presenting with fever.

Equal distribution of sex of the children presenting with fever is naturally expected as was found in our study (Table 3). Almost equal distribution of sex was reported in other studies [7, 20]. Most children with SBIs were in the 3–12 months age group (Table 3). Similar findings were reported in an Indian study, which reported 77% of the children with fever were infants [7].

None of the patients in diagnosed as bronchiolitis had SBIs in the present study. Low percentages of SBIs in children with viral pneumonia/bronchiolitis has been reported [20]. Most diagnostic algorithms classify infants with infections caused by respiratory syncytial and influenza viruses as low risk for SBI, including UTI (5.6%) and bacteremia (1.4%) [21]. On the other hand most children with radiologically confirmed bacterial pneumonia have a confirmed SBI [22]. In our study, out of the 38 patients presenting with UTI, only 9 (24%) had confirmed SBI. A low (18%) [19] and high (30%) [22] proportion of SBI in UTI in febrile children have been reported. UTI remains the most common cause of non-localized fever in children below 2 years of age and can be a co-infection in confirmed viral infections as well [21]. Therefore, urine analysis and culture should remain a standard part diagnostic work-up of infants and toddlers with fever. In our study, 29/31 (94%) of the children presenting with severe acute malnutrition (SAM) and fever had confirmed SBI with majority (86%) having bronchopneumonia. A high proportion of bronchopneumonia responsible for SBI in patients with SAM has been reported by other studies from India, Zambia, and East Africa [23–25]. An East African study reported a higher rate of sepsis in patients with SAM than that in the present study [23]. The synergism between malnutrition and infectious disease is well known. Protein and vitamin deficiency inhibit the formation of specific antibodies and also cause impairment of the pulmonary defense mechanism [26]. The increased incidence and severity of infections in malnourished children is due to limited production and diminished functional capacity of B-cell and T-cell components of the immune system [27]. In our case series, of the 25 patients with simple febrile convulsion, only 1 (4%) had confirmed SBI. The results confirm a low risk for SBI in first-time seizures in children [20].

The diagnosis of enteric fever is a major concern in low-and-middle-income countries including our settings. The main diagnostic problem is that the yield of gold standard-blood culture is a low 25% (*n* = 10/40) in the present study. Blood cultures are usually not available in many low-income settings. Low rates of culture positivity (19%) in suspected enteric fever has been reported from other tertiary care hospitals in India [8]. The main cause for such low rates is antibiotic use prior to culture [8]. Widal often is the only test available for diagnosis in low-middle-income countries. Therefore, if we included Widal test in our diagnostic panel with a higher 1: 320 dilution threshold than the suggested 1:160 dilution for agglutinating antibodies to O and H antigens of *Salmonella typhi* [28]. This helped us in diagnosing an additional 12 enteric fever cases with SBI (Table 1). Thus, Widal can be used in appropriate clinical setting to diagnose probable cases, with the caveat that the test might be false positive in malaria, dengue and disseminated tuberculosis [28].

Most patient with culture positive bacterial infections including SBI usually require a longer hospital stay for completion of antibiotics and recovery from other co-morbidities. This fact has been reported in our settings [29] and also in other settings [20]. A history of previous hospitalization increased the risk for SBI 15-fold (Table 2). Another study reported a 3-fold increase in the risk of SBI in patients with a history of hospitalization [30]. Furthermore, SBI risk in chronic diseases, such as tuberculosis, HIV infection, and diabetes, was reported [7]. As observed in this study, the increased risk of SBI in partially immunized or unimmunized children compared with fully immunized children have been reported earlier [7, 20]. Although not specifically evaluated in this study increasing use of Hib vaccine and more recently PCV will further reduce the risk for SBI [31]. In our study, on general examination, we observed that 83% of the children who had bacterial infection had no BCG scar mark. Another Indian study [7] observed that 60% of the children with bacterial infection were without BCG scar mark. Recent evidence suggests that the effect of BCG vaccine extends beyond the target-disease immunity [32]. These non-specific effects of BCG are due to epigenetic reprogramming of innate immune cells termed “trained immunity”. Numerous studies demonstrate that BCG vaccination impacts the immune response to subsequent infections, resulting in reduced morbidity and mortality, especially viral infections [31].

Our study found that children with developmental delay had increased risk of SBI. A study showed that 70% of the children with developmental delay had SBI [33]. Possible reasons could be a lack of verbal ability in developmentally delayed children and nonspecific nature of presenting complaints of SBI.

Children with bacterial infections presented common symptoms, such as breathlessness in 80%, poor feeding in 75%, irritability in 75%, weight loss in 92%, suspected UTI in 79%, and common cold in 71% patients (Table 4). A high prevalence (50–64%) of respiratory symptoms in children with SBI has been reported [33]. Up to 78% of febrile children reported SBI with poor feeding and 76% children reported SBI with irritability [30]. Suspected UTI was reported as the main presenting complaint in approximately 70% of the patients diagnosed with SBI [30]. A “sick-looking/toxic looking” child is a common general examination finding in children with confirmed bacterial infections [18, 33, 34]. Thus, if a febrile child is considered “sick looking” by a pediatrician, such a child should be investigated even if no other clues to a SBI are present.

Hyponatremia, hypokalemia, and hypocalcemia were reported in sick children [35]. However, studies reporting SBI and electrolyte abnormalities are lacking [36]. Abnormal leucocyte counts have been reported in other studies as an indicator for SBI [20, 37].

Common pathogens identified in the present study are similar to those reported in other studies [5, 8, 38–42]. However, the prevalence of extended-spectrum beta-lactamase and multidrug-resistant pathogens was high but should be interpreted with caution in view of the small sample size. The problem of antimicrobial resistance has been documented in many settings including India, where many reports of high prevalence of antibiotic-resistant bacteria have emphasized commensal bacteria [43] as well as pathogenic bacteria [40]. We hope that introduction of more diagnostic tools especially point-of-care diagnostics will reduce irrational antibiotic use.

### Limitations

We did not investigate viral, parasitic (except malaria) and fungal co-infections, due to limited laboratory and financial capacity. We used blood, CSF and urine cultures to delineate the bacterial aetiology. Respiratory sample were not cultured. We did not do microscopic stool examination during the study, which could have helped to identify few parasitic infections. For pneumonia we used radiological defined opacity as well as CRP value, which were not validated in Indian children at the time of the study. However, we feel that adding CRP cut-offs to radiological defined opacity will reduce irrational antibiotic use.

## Conclusions

The study identified multiple risk factors for SBI. Pediatricians can be made aware of these risk factors. The results of this study should help develop clinical prediction rules that can supplement the current clinical practice guidelines. Further studies are warranted to identify age-specific risk factors for SBI as most clinicians depend on clinical signs and symptoms to identify SBIs.

## Supplementary information

**Additional file 1: Table S1.** Definitions [44, 45].

**Additional file 2: Table S2.** Spectrum of activity of antimicrobials against five most prevalent causes of Gram-positive infections in study, Ujjain, India.

**Additional file 3: Table S3.** Spectrum of resistance* of antimicrobials against four most prevalent causes of Gram-negative infections in study, Ujjain, India.

## Data Availability

The datasets used and/or analysed during the current study is available from the corresponding author on reasonable request.

## Abbreviations

CI: Confidence intervals
CRP: C-reactive protein
ROC: Receiver-operating-characteristics curves
RR: Risk ratios
SAM: Severe acute malnutrition
SBI: Serious bacterial infection
UTI: Urinary tract infection
