# Supplementary material for: Incidence, clinical profile, and risk factors for serious bacterial infections in children hospitalized with fever in Ujjain, India
Source: BMC Infect Dis. 2020 Feb 21;20:162. doi: 10.1186/s12879-020-4890-6 (PMC7035762; doi:10.1186/s12879-020-4890-6)
Supplement: Supplementary file 1 — Additional file 1: Table S1. Definitions [44, 45]. [file 12879_2020_4890_MOESM1_ESM.docx]

**Table S1: Definitions**

| Condition/term | Definition | Ref. no. |
| --- | --- | --- |
| Socioeconomic classification | Modified Kuppuswamy classification (details in reference) | [[44](#_ENREF_44)] |
| Delayed development | A child was said to have delayed development if after assessment the child was delayed for age and sex in any of gross motor, fine motor, language, or social domain | [[3](#_ENREF_3)] |
| Significant lymphadenopathy | Significant lymphadenopathy was considered if size of inguinal nodes was more than1.5cm in diameter, and/or cervical /axillary more than 1cm in diameter and/or on multiple sites, and/or presence of matted nodes, and/or if lymphadenopathy was associated with focus of infection, and/or with systemic signs and symptoms | [[3](#_ENREF_3)] |
| Severe acute malnutrition (SAM) | Severe acute malnutrition (SAM) was assessed in children between the ages of 6 month to 5 years according to Indian Academy of Pediatrics consensus statement for diagnosis and management of SAM | [[45](#_ENREF_45)] |
| Partial immunization | A child that is incompletely immunized for age |  |
| Suspected urinary tract infection | Suspected urinary tract infection was defined as presenting complaints of burning micturition, pain in abdomen, frequency, urgency and urine microscopy done on a spun urine sample within two hours of collection, showing more than 5 white blood cells per high power field or a child below 2 years of age presenting with fever without identifiable source of infection and microscopy revealed >5 leucocytes more than 5 white blood cells per high power field on a spun urine sample | [[3](#_ENREF_3)] |
| Poor feeding | Poor feeding was defined as when an infant has lack of interest in feeding or a problem receiving proper amount of nutrition. | [[3](#_ENREF_3)] |
| Weight loss | Weight loss was defined as loss of >10% of the actual weight of the individual during a period of 1 month and was labeled only if the weight was documented | [[3](#_ENREF_3)] |
| Febrile seizures | Simple febrile seizure was a primary generalized, usually tonic-clonic attack associated with fever with seizures lasting for maximum of 15 minutes and did not recur within 24-hour period | [[3](#_ENREF_3)] |
| Electrolyte abnormalities | Hyponatremia and hypernatremia were defined as serum sodium concentration below 130 mEq/L and above 150 mEq/L, respectively. Hypokalemia and hyperkalemia were defined as potassium level below 3.5 mEq/L and above 5.5 mEq/L, respectively | [[3](#_ENREF_3)] |
| Abnormal leucocyte count | Abnormal leucocyte count was defined as a leucocyte count in a child up-to 2 year of age <6000 cells/mm^3^ and/or >14000 cells/mm^3^. In a child >2year of age, <4000 cell/mm^3^ and/or >12000 cells/mm^3^ | [[3](#_ENREF_3)] |
| Septicemia | Clinical syndrome characterized by fever, chills, malaise,  tachycardia, etc. when circulating bacteria multiply at a rate that exceeds removal by phagocytosis | [[3](#_ENREF_3)] |
