# Supplementary material for: Incidence, clinical profile, and risk factors for serious bacterial infections in children hospitalized with fever in Ujjain, India
Source: BMC Infect Dis. 2020 Feb 21;20:162. doi: 10.1186/s12879-020-4890-6 (PMC7035762; doi:10.1186/s12879-020-4890-6)
Supplement: Supplementary file 2 — Additional file 2: Table S2. Spectrum of activity of antimicrobials against five most prevalent causes of Gram-positive infections in study, Ujjain, India. [file 12879_2020_4890_MOESM2_ESM.docx]

**Table S2:** Spectrum of activity of antimicrobials against five most prevalent causes of Gram-positive infections in study, Ujjain, India

|  | MSSA (n = 10) | | | MRSA (n = 7) | | | S. pneumonia (n = 7) | | | E. faecalis (n = 6) | | | S. epidermidis (n = 2) | | |
| --- | --- | --- | --- | --- | --- | --- | --- | --- | --- | --- | --- | --- | --- | --- | --- |
|  | R (%) | IR (%) | S (%) | R (%) | IR (%) | S (%) | R (%) | IR (%) | S (%) | R (%) | IR (%) | S (%) | R (%) | IR (%) | S (%) |
| **Penicillin** |  |  |  |  |  |  |  |  |  |  |  |  |  |  |  |
| Penicillin | 90 | 10 | 0 | 100 | 0 | 0 | 28 | 0 | 72 | - | - | - | 100 | 0 | 0 |
| Oxacillin | 0 | 0 | 100 | 100 | 0 | 0 | 72 | 14 | 14 | - | - | - | - | - | - |
| Ampicillin | - | - | - | - | - | - | - | - | - | 33 | 0 | 67 | - | - | - |
| **Cephalosporins** |  |  |  |  |  |  |  |  |  |  |  |  |  |  |  |
| Cefoxitin | 0 | 20 | 80 | 100 | 0 | 0 | 80 | 0 | 20 | - | - | - | 50 | 0 | 50 |
| **Quinolone** |  |  |  |  |  |  |  |  |  |  |  |  |  |  |  |
| Ciprofloxacin | 50 | 10 | 40 | 72 | 0 | 28 | 100 | 0 | 0 | 83 | 0 | 17 | 0 | 0 | 100 |
| **Macrolides-lincosamides** |  |  |  |  |  |  |  |  |  |  |  |  |  |  |  |
| Erythromycin | 20 | 10 | 70 | 72 | 14 | 14 | 72 | 0 | 28 | - | - | - | 50 | 0 | 50 |
| Clindamycin | 10 | 0 | 90 | 14 | 0 | 86 | 14 | 0 | 86 | - | - | - | 50 | 0 | 50 |
| Vancomycin | 0 | 0 | 100 | 0 | 0 | 100 | 0 | 0 | 100 | 0 | 0 | 100 | 0 | 0 | 100 |
| **Other classes** |  |  |  |  |  |  |  |  |  |  |  |  |  |  |  |
| Lenezolid | 30 | 0 | 70 | 28 | 0 | 72 | 30 | 0 | 70 | 33 | 0 | 67 | 50 | 0 | 50 |
| Tetracycline | 40 | 0 | 60 | 72 | 0 | 18 | 44 | 0 | 54 | - | - | - | 0 | 50 | 50 |
| Teicoplanin | 0 | 10 | 90 | 0 | 0 | 100 | 14 | 0 | 86 | 0 | 0 | 100 | 0 | 0 | 100 |
| Tigecycline | 0 | 0 | 100 | 0 | 0 | 100 | 14 | 0 | 86 | - | - | - | 50 | 0 | 50 |
| Trimethoprim-sulfamethoxazole | 40 | 0 | 60 | 44 | 0 | 56 | 72 | 14 | 14 | - | - | - | 50 | 0 | 50 |
| Gentamicin | - | - | - | - | - | - | 72 | 14 | 14 | 33 | 0 | 77 | - | - | - |

R-resistance, IR-Intermediate resistance, S-sensitive
