# Supplementary material for: Incidence, clinical profile, and risk factors for serious bacterial infections in children hospitalized with fever in Ujjain, India
Source: BMC Infect Dis. 2020 Feb 21;20:162. doi: 10.1186/s12879-020-4890-6 (PMC7035762; doi:10.1186/s12879-020-4890-6)
Supplement: Supplementary file 3 — Additional file 3: Table S3. Spectrum of resistance* of antimicrobials against four most prevalent causes of Gram-negative infections in study, Ujjain, India. [file 12879_2020_4890_MOESM3_ESM.docx]

Table S3: Spectrum of resistance* of antimicrobials against four most prevalent causes of Gram-negative infections in study, Ujjain, India

|  | Total culture positive Gram-negative infections n=60 | | | | | | |
| --- | --- | --- | --- | --- | --- | --- | --- |
| **Percentage resistance** | *E. coli* (n=25) | | *P. aeruginosa* (n=17) | | *S.* Typhi (n=10) | *K. pneumoniae* (n=5*)* | *A. baumannii* (n=3) |
|  | Blood + CSF | Urine | Blood + CSF | Urine | Blood | Blood | Blood |
|  | (n=15+3) | (n=7) | (n=13+2) | (n=2) | (n=10) | (n=5) | (n=3) |
| **Penicillin** |  |  |  |  |  |  |  |
| Ampicillin | 73% | 71% | - | - | 100% | - | - |
| Piperacillin/tazobactam | 67% | - | 73% | 50% | - | 40% | 67% |
| **Cephalosporins** |  |  |  |  |  |  |  |
| Cefixime | - | - | - | - | 20% | - | - |
| Ceftazidime | 73% | - | 73% | 50% | - | 20% | 100% |
| Ceftriaxone | 80% | - | - | - | 20% | - | - |
| Cefipime | 20% | - | 33% | 50% | - | - | 67% |
| Cefotaxime | 73% | 57% | - | - | - | 20% | - |
| Cefazolin | - | 57% | - | - | - | - | - |
| **Other β lactam** |  |  |  |  |  |  |  |
| Imipenem | 27% | 28% | 33% | 50% | - | 60% | 100% |
| Ertapenem | 20% | 14% | - | - | - | 40% | - |
| **Quinolones** |  |  |  |  |  |  |  |
| Ciprofloxacin | 60% | 57% | 66% | 100% | 40% | 40% | - |
| **Aminoglycosides** |  |  |  |  |  |  |  |
| Gentamicin | 80% | - | 40% | 50% | - | - | - |
| Amikacin | 40% | 43% | 33% | 50% | - | 40% | 67% |
| **Others** |  |  |  |  |  |  |  |
| Cotrimoxazole | 80% | 86% | - | - | 50% | - | - |
| Nitrofurantoin | - | 14% | - | - | - | - | - |
| Levofloxacin | 20% | 43% | 27% | 100% | - | 40% | 67% |
| Azithromycin | - | - | - | - | 20% | - | - |
| Chloramphenicol | - | - | - | - | 20% | - | - |
| Colistin | 7% | 14% | 7% | 50% | - | 80% | - |
| Tobramycin | - | - | 7% | 50% | - | - | - |

*Resistant isolates include intermediate resistant isolates
